# Supplementary material for: Triggering of the immune response to MCF7 cell line using conjugated antibody with bacterial antigens: In-vitro and in-vivo study
Source: PLoS One. 2022 Oct 7;17(10):e0275776. doi: 10.1371/journal.pone.0275776 (PMC9543947; doi:10.1371/journal.pone.0275776)
Supplement: S3 File — (DOCX) [file pone.0275776.s003.docx]

Fig 5 data:

| Groups | MCF7 | HUVEC |
| --- | --- | --- |
| Ab 2μg/ml | 87.10±3.95 | 100.46±0.001 |
| Ab 4μg/ml | 83.14±6.91 | 100.32±0.001 |
| Ab-Ag 4μg/m | 88.1400±7.6 | 100.00000 |
| Ab-Ag 8μg/m | 90.33±3.69 | 100.73±0.001 |
| Ab-Ag 16μg/m | 93.80±1.63 | 93.56±0.001 |
| Ab-Ag 30μg/m | 84.41±12.2 | 93.76±0.95 |
| Ab-Ag 50μg/m | 84.01±7.33 | 92.62±14.46 |

Fig 6 data:

| Groups | MCF7 | HUVEC |
| --- | --- | --- |
| Control | 100 | 100 |
| Active Human Serum 40% | 88.47±15.25 | 94.59±8.69 |
| Ab-Ag 4μg/m | 88.17 | 100 |
| Ab-Ag 4μg/m +Active Human Serum | 59.74±5.31 | 106.49±5.23 |
| Ab-Ag 8μg/m | 90.33±3.69 | 109.73±0.001 |
| Ab-Ag 8μg/m +Active Human Serum | 61.18±5.49 | 92.13±2.15 |
| Ab-Ag 16μg/m | 93.80±1.63 | 93.56±0.001 |
| Ab-Ag 16μg/m +Active Human Serum | 62.02±9.03 | 80.80±11.59 |
| Ab-Ag 30μg/m | 84.01±12.2 | 93.76±0.95 |
| Ab-Ag 30μg/m +Active Human Serum | 61.16±2.93 | 78.24±30.25 |
| Ab-Ag 50μg/m | 84.40±7.33 | 92.62±14.46 |
| Ab-Ag 504μg/m +Active Human Serum | 55.92±7.62 | 79.02±19.88 |

Fig 9 data:

| Groups | MCF7 | HUVEC |
| --- | --- | --- |
| Control | 100 | 100 |
| Dox 0.5μg/m | 66.40±10.61 | 43.95±4.5 |
| Dox 1μg/m | 38.42±6.35 | 34.70±2.63 |
| Dox 2μg/m | 35.34±4.03 | 30.47±2.57 |
| Ab-Dox 1μg/ml | 70.61±1.641 | 87.01±2.94 |
| Ab-Dox 2μg/ml | 58.84±5.77 | 71.83±3.71 |
| Ab-Dox 3μg/ml | 55.62±5.84 | 62.76±10.76 |
| Ab-Dox 4μg/ml | 51.46±7.98 | 53.19±4.38 |
| Ab-Dox 5μg/ml | 36.19±5.08 | 46.3±2.38 |
