# Supplementary figures and images for: Triggering of the immune response to MCF7 cell line using conjugated antibody with bacterial antigens: In-vitro and in-vivo study
Source: PLoS One. 2022 Oct 7;17(10):e0275776. doi: 10.1371/journal.pone.0275776 (PMC9543947; doi:10.1371/journal.pone.0275776)

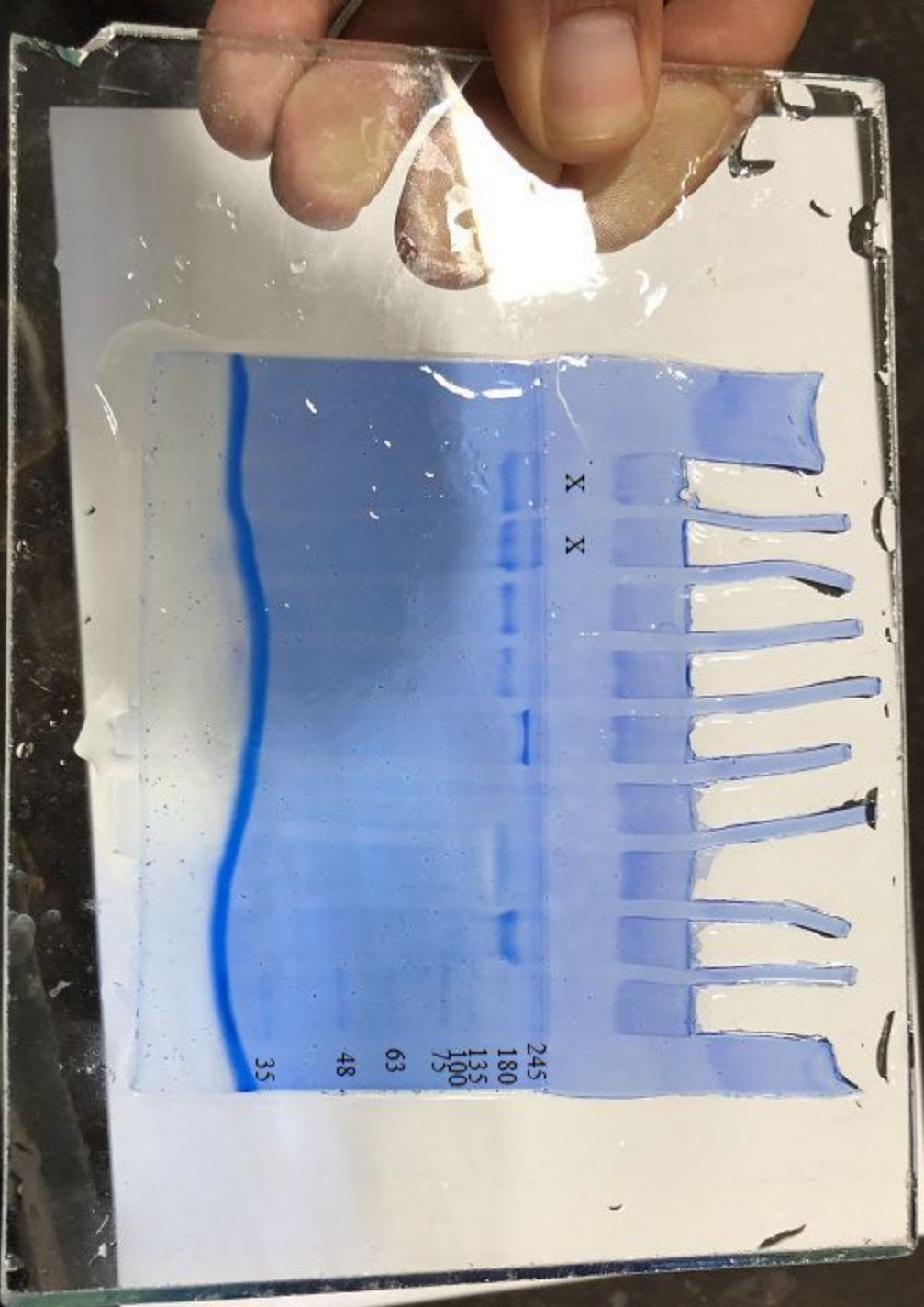

Supplement: S1 Raw images — (PDF) [file pone.0275776.s005.pdf]
