# Supplementary material for: Triggering of the immune response to MCF7 cell line using conjugated antibody with bacterial antigens: In-vitro and in-vivo study
Source: PLoS One. 2022 Oct 7;17(10):e0275776. doi: 10.1371/journal.pone.0275776 (PMC9543947; doi:10.1371/journal.pone.0275776)
Supplement: Attachment — Submitted filename: Response to reviewers.docx [file pone.0275776.s006.docx]

Dear reviewers and academic editor,

Thank you for your review of the manuscript. According to valuable comments the manuscript entitled **Triggering of the immune response to MCF7 cell line using conjugated antibody with bacterial antigens: In-vitro and In-vivo study** was revised. In the following sections, you will find the responses to each of the points and suggestions. We are grateful for the time you expended on our behalf.

With the best regards

**Additional requirements**

1. Please ensure that your manuscript meets PLOS ONE's style requirements.

The manuscript was checked and matched to PLOS ONE format.

2. To comply with PLOS ONE submissions requirements, in your Methods section, please provide additional information regarding the experiments involving animals and ensure you have included details on (1) methods of sacrifice, (2) methods of anesthesia and/or analgesia, and (3) efforts to alleviate suffering.

All of the mentioned requirements, were stated in "Ethics Statement**"** section**.**

3. We note that the grant information you provided in the ‘Funding Information’ and ‘Financial Disclosure’ sections do not match.

Our research project was financially supported by Shahid Chamran University of Ahvaz, Iran, under the grant number of 98/3/05/14909.

4. Thank you for stating the following in the Acknowledgments Section of your manuscript:

"This study was financially supported by Shahid Chamran University of Ahvaz, Ahvaz, Iran, under the grant number of 98/3/05/14909. …

Please include your amended statements within your cover letter; we will change the online submission form on your behalf.

The statement was moved to cover letter.

5. We note that you have indicated that data from this study are available upon request. PLOS only allows data to be available upon request if there are legal or ethical restrictions on sharing data publicly.

b) If there are no restrictions, please upload the minimal anonymized data set necessary to replicate your study findings as either Supporting Information files or to a stable, public repository and provide us with the relevant URLs, DOIs, or accession numbers. For a list of acceptable repositories, please see http://journals.plos.org/plosone/s/data-availability#loc-recommended-repositories

The supporting information was provided as will submitted along with revised manuscript.

6. PLOS ONE now requires that submissions reporting blots or gels include original, uncropped blot/gel image data as a supplement or in a public repository.

The uncropped image of the SDS-PAGE gel will submitted along with the revised manuscript.

7. Please amend your list of authors on the manuscript to ensure that each author is linked to an affiliation.

The authors was linked to the affiliations.

8. Please amend either the abstract on the online submission form (via Edit Submission) or the abstract in the manuscript so that they are identical.

The abstract will be adjusted in manuscript and online form.

**Reviewers' comments**

1. Please do change the manuscript tittle to: Triggering of the immune response against MCF7 cell line using conjugated antibody with bacterial antigens: In-vitro and In-vivo study

Thanks for your suggestion; the tittle replaced as you noted.

2. In general, there are some typo errors and grammar mistake. Please make sure about them.

The language of the manuscript checked for grammar and dictation mistakes; the corrected words and sentences highlighted in red. Thanks for your valuable comments.

3. Please rewrite the main finding in abstract and conclusion sections to be clear and readable for readers in future. (Do focus on novelty of your work)

The main finding in abstract and conclusion sections were revised as you noted about novelty of the research.

4. The Introduction section need to improve. In introduction, you have to write sufficient background information, and the purpose of the article is clearly defined at the end of the introduction part.

The introduction section was revised according to the comment.

5. Most of materials and methods section needed to add references.

The references were added to the methods. The new references was highlighted in red.

6. In figures legends of figure 2 you wrote: Figure 2. Figure 1. The Attenuated Total Reflection Fourier-Transform Infrared 537 Spectroscopy (ATR-FTIR) analysis of the antibodies conjugated to doxorubicin (Ab-Dox) 538 and bacterial antigens (B-Ab), purified anti-MCF7 antibodies (Ab) and doxorubicin (Dox). Please do remove Figure 1 from sentence.

This mistake was corrected. Thanks for your valuable comments.

7. Please do change figure 4 legend from The control cell line of MCF7 (row 1) and HUVEC cell lines (row 2). To Morphology of the control cell line of MCF7 (row 1) and HUVEC cell lines (row 2).

The legend was corrected as mentioned. Thanks for your valuable comments.

8. Please do change figure 11 legend from Histological slides of the grafted MCF7 scaffolds; recruitment of the inflammatory (black arrows) and fibroblast cells (white arrows) occurred at different proportions. To Histological changes of the grafted MCF7 scaffolds; recruitment of the inflammatory (black arrows) and fibroblast cells (white arrows) occurred at different proportions.

The legend was corrected as mentioned. Thanks for your valuable comments.
